# A computationally efficient clustering linear combination approach to jointly analyze multiple phenotypes for GWAS

**DOI:** 10.1371/journal.pone.0260911

**Published:** 2022-04-28

**Authors:** Meida Wang, Shuanglin Zhang, Qiuying Sha

**Affiliations:** Mathematical Sciences, Michigan Technological University, Houghton, MI, United States of America; Massachusetts General Hospital/Harvard Medical School, UNITED STATES

## Abstract

There has been an increasing interest in joint analysis of multiple phenotypes in genome-wide association studies (GWAS) because jointly analyzing multiple phenotypes may increase statistical power to detect genetic variants associated with complex diseases or traits. Recently, many statistical methods have been developed for joint analysis of multiple phenotypes in genetic association studies, including the Clustering Linear Combination (CLC) method. The CLC method works particularly well with phenotypes that have natural groupings, but due to the unknown number of clusters for a given data, the final test statistic of CLC method is the minimum p-value among all p-values of the CLC test statistics obtained from each possible number of clusters. Therefore, a simulation procedure needs to be used to evaluate the p-value of the final test statistic. This makes the CLC method computationally demanding. We develop a new method called computationally efficient CLC (ceCLC) to test the association between multiple phenotypes and a genetic variant. Instead of using the minimum p-value as the test statistic in the CLC method, ceCLC uses the Cauchy combination test to combine all p-values of the CLC test statistics obtained from each possible number of clusters. The test statistic of ceCLC approximately follows a standard Cauchy distribution, so the p-value can be obtained from the cumulative density function without the need for the simulation procedure. Through extensive simulation studies and application on the COPDGene data, the results demonstrate that the type I error rates of ceCLC are effectively controlled in different simulation settings and ceCLC either outperforms all other methods or has statistical power that is very close to the most powerful method with which it has been compared.

## Introduction

Genome-wide association study (GWAS) has successfully identified a large number of genetic variants that are associated with human complex diseases or phenotypes [1–4]. Among these results, a phenomenon in which a genetic variant affects multiple phenotypes often occurs [5], which is significant evidence to show that pleiotropic effects on human complex diseases are universal [6–9]. Moreover, several disease-related phenotypes are usually measured simultaneously as a disorder or risk factors of a complex disease in GWAS. Therefore, considering the correlated structure of multiple phenotypes in genetic association studies can aggregate multiple effects and increase the statistical power [10–15].

At present, a variety of approaches that focus on jointly analyzing multiple phenotypes have been proposed. These statistical methods can be roughly divided into three categories, including approaches based on regression models [16–19], combining the univariate analysis results [20–23], and variable reduction techniques [24–27]. For example, MultiPhen [19] performs an ordinal regression model, which uses an inverted model whereby the phenotypes are the predictor variables and the genotype is the dependent variable [28, 29]. In terms of the second category, combining the univariate test statistics or integrating the p-values of univariate tests are two basic methods. For instance, the O’Brien [20, 21] method constructs a test statistic for pleiotropic effect by combining univariate test statistics of multiple phenotypes; the Trait-based Association Test that uses the Extended Simes procedure (TATES) [23] integrates the p-values from univariate tests to obtain an overall trait-based p-value. In addition, principal components analysis of phenotypes (PCP) [24], principal component of heritability (PCH) [25, 26], and canonical correlation analysis (CCA) [27] are three variable reduction methods in the third category. Furthermore, with more and more GWAS summary statistics from univariate phenotype analysis in the traditional GWAS being publicly available, many approaches, such as MTAG [30], CPASSOC [31], and MPATs [32] that are only based on the GWAS summary statistics, were proposed.

In practice, multiple phenotypes considered may be in different clusters, but most methods for detecting the association between multiple phenotypes and genetic variants either treat all phenotypes as a group or treat each phenotype as one group and combine the results of univariate analysis. Unlike these methods, the clustering linear combination (CLC) method [33] works particularly well with phenotypes that have natural clusters. In the CLC method, individual statistics from the association tests for each phenotype are clustered into positively correlated clusters using the hierarchical clustering method, then the CLC test statistic is used to combine the individual test statistics linearly within each cluster and combine the between-cluster terms in a quadratic form. It was theoretically proved that if the individual statistics can be clustered correctly, the CLC test statistic is the most powerful test among all tests with certain quadratic forms [33]. Due to the unknown number of clusters for a given data, the final test statistic of CLC method is the minimum p-value among all p-values of the CLC test statistics obtained from each possible number of clusters. Therefore, a simulation procedure needs to be used to evaluate the p-value of the final test statistic because it does not have an asymptotic distribution, and that makes the CLC method computationally demanding. If we can construct a test statistic with an approximate distribution, the computational efficiency will be greatly improved. In this paper, based on the Aggregated Cauchy Association Test (ACAT) method [34], we develop a new method named computationally efficient CLC (ceCLC). In ceCLC, the p-values of the CLC test statistics with *L* clusters are transformed to follow a standard Cauchy distribution, then the transformed p-values are combined linearly with equal treatment to obtain the ceCLC test statistic. This test statistic of ceCLC has an approximately standard Cauchy distribution even though there is a correlated structure between combined p-values [35], so the p-value of the ceCLC test statistic can be calculated based on the cumulative density function of standard Cauchy distribution. We perform extensive simulation studies and apply ceCLC to the COPDGene real dataset. The results show that the ceCLC method has correct type I error rates and either outperforms all other methods or has statistical power that is very close to the most powerful method with which it has been compared.

## Materials and methods

Assume we consider *N* unrelated individuals with *K* correlated phenotypes, which can be quantitative or qualitative (binary), and each individual has been genotyped at a genetic variant of interest. Let *Y*_*i*_ = (*Y*_*i*1_,⋯,*Y*_*iK*_)^*T*^ represent *K* correlated phenotypes for the *i*th individual (1 for cases and 0 for controls for a qualitative trait) with *i* = 1,2,⋯,*N*. Let *G*_*i*_ denote the genotype for the *i*th individual at the variant of interest, where *G*_*i*_∈{0, 1, 2} corresponds to the number of minor alleles. We suppose that there are no covariates. If there are *p* covariates *z*_*i*1_,…,*z*_*ip*_, we adjust both genotypes and phenotypes for the covariates [36, 37] using linear models $G_{i}=\alpha_{0}+\alpha_{1}z_{i1}+\cdots +\alpha_{p}z_{ip}+\varepsilon_{i}$ and $Y_{ik}=\alpha_{0k}+\alpha_{1k}z_{i1}+\cdots +\alpha_{pk}z_{ip}+\tau_{ik}$, and use the residuals of the respective linear models to replace the original genotypes and phenotypes.

For each phenotype, we consider the following generalized linear model [38]:

$$g(E(Y_{ik}|G_{i}))=\beta_{0k}+\beta_{1k}G_{i},$$

where *β*_1*k*_ is the genetic effect of the variant on the *k*th phenotype and g(∙) is a monotone “link” function. Two types of generalized linear model are commonly used: 1) linear model with an identity link for quantitative phenotypes and 2) logistic regression model with a logit link for qualitative phenotypes. We first conduct a univariate test to test *H*_0_: *β*_1*k*_ = 0 for each phenotype, *k* = 1,2,⋯,*K*, using the score test statistic [39]

$$T_{k}=U_{k}/\sqrt{V_{k}},$$

where $U_{k}=\sum_{i=1}^{N}Y_{ik}(G_{i}-\bar{G})$ and $V_{k}=\frac{1}{N}\sum_{i=1}^{N}{\left(Y_{ik}-{\bar{Y}}_{k}\right)}^{2}\sum_{i=1}^{N}{\left(G_{i}-\bar{G}\right)}^{2}$. Since the test statistic *T*_*k*_ has an approximate normal distribution with mean *μ*_*k*_ = *E*(*T*_*k*_) and variance 1, we can assume that *T* = (*T*_1_,⋯,*T*_*K*_)^*T*^ approximately follows a multivariate normal distribution with mean vector *μ* = (*μ*_1_,⋯,*μ*_*K*_)^*T*^ and covariance matrix Σ. Our objective is to test the association between multiple phenotypes and a genetic variant, so the null hypothesis is *H*_0_: *β*_11_ = ⋯ = *β*_1*K*_ = 0. Sha et al. [33] showed that under the null hypothesis, Σ converges to *P*(*Y*) almost surely, where *P*(*Y*) is the correlation matrix of *Y* = (*Y*_1_,⋯,*Y*_*K*_)^*T*^. Therefore, we can use the sample correlation matrix of *Y*, *P*^*s*^(*Y*), to estimate Σ.

Based on the CLC [33] and ACAT methods [34], we propose a computational efficient CLC (ceCLC) method in this paper. Same as the CLC method [33], we use the hierarchical clustering method with similarity matrix $\hat{\Sigma }=P^{s}(Y)$ and dissimilarity matrix 1−*P*^*s*^(*Y*) to cluster *K* phenotypes. Suppose that the phenotypes are clustered into *L* clusters, considering *L* = 1,⋯,*K*, and *B* is a *K*×*L* matrix with the (*k*, *l*)^*th*^ element equals 1 if the *k*th phenotype belongs to the *l*th cluster, otherwise it equals 0. The CLC test statistic [33] with *L* clusters is given by

$$T_{CLC}^{L}={\left(WT\right)}^{T}{\left(W\Sigma W^{T}\right)}^{-1}(WT),$$

where $W=B^{T}\Sigma^{-1}.\;T_{CLC}^{L}$ follows a $\chi_{L}^{2}$ distribution under the null hypothesis, therefore we can obtain the p-value of $T_{CLC}^{L}$, represented by *p*_*L*_, for *L* = 1,⋯,*K*. Since for a given data set, the number of clusters of the phenotypes is unknown, in the last step of the CLC method [33], *T*_*CLC*_ = min_1≤*L*≤*K*_ *p*_*L*_ is used as the final test statistic. Because *T*_*CLC*_ does not have an asymptotic distribution, a simulation procedure is needed to evaluate the p-value of *T*_*CLC*_. This makes the CLC method computationally demanding. In this paper, instead of using the minimum p-value as the test statistic in the CLC method, we use the Cauchy combination test [35] to combine all p-values of the CLC test statistics obtained from each possible number of clusters. We define the ceCLC test statistic as the linear combination of the transformed p-values over the number of *K* clusters, which is given by

$$T_{ceCLC}=\frac{1}{K}\sum_{L=1}^{K}tan\{(0.5-p_{L})\pi \}$$

Under the null hypothesis, we know that *p*_*L*_ is uniformly distributed between 0 and 1, therefore tan {(0.5−*p*_*L*_)*π*} follows a standard Cauchy distribution. If *p*_1_,⋯,*p*_*K*_ are independent, the test statistic *T*_*ceCLC*_ follows a standard Cauchy distribution under the null hypothesis. However, most likely there exists a correlated structure between *p*_1_,⋯,*p*_*K*_. Liu. et. al [35] has proved that a weighted sum of “correlated” standard Cauchy variables still has an approximately Cauchy tail, and the influence of correlated structure on the tail is quite limited because of the heaviness of the Cauchy tail. Therefore, *T*_*ceCLC*_ can be well approximated by a standard Cauchy distribution. According to the cumulative density distribution of standard Cauchy distribution, the p-value of *T*_*ceCLC*_ can be approximated by 0.5−{arctan(*T*_*ceCLC*_)/*π*}. The R code for the implementation of ceCLC is available at github https://github.com/MeidaWang/ceCLC.

## Results

### Simulation design

In our simulation studies, we generate one common variant and *K* = 20 and 40 correlated phenotypes for *N* individuals. Firstly, we generate the genotypes of the genetic variant according to the minor allele frequency (MAF = 0.3) under Hardy Weinberg equilibrium. Secondly, the *K* quantitative phenotypes are generated by the following factor model [22, 26, 28, 33]

$$Y=\lambda G+c\gamma f+\sqrt{1-c^{2}}\times \varepsilon .$$

where *Y* = (*Y*_1_,⋯,*Y*_*K*_)^*T*^, *G* is the genotype at the variant of interest, *λ* = (*λ*_1_,⋯,*λ*_*K*_)^*T*^ is the vector of genetic effect sizes on *K* phenotypes, *c* is a constant number, *f* is a vector of factors, and $f={\left(f_{1},\cdots ,f_{R}\right)}^{T}∼MVN(0,\Sigma )$, where *R* is the number of factors, Σ = (1−*ρ*)*I*+*ρA*, all elements of matrix *A* equals 1, *I* is an identity matrix, *ρ* is the correlation between factors; *γ* is a *K*×*R* matrix, *ε* = (*ε*_1_,⋯,*ε*_*K*_)^*T*^ is a vector of residuals, and *ε*_1_,⋯,*ε*_*K*_~i.i.d. *N*(0,1).

According to different number of factors affected by the genotypes and different effect sizes, we consider the following four models. In each model, the within-factor correlation is *c*^2^ and the between-factor correlation is *ρc*^2^. We set *c* = 0.5 and *ρ* = 0.6.

Model 1: There is only one factor and genotypes influence all phenotypes. That is, *R* = 1, *λ* = *β*(1,2,⋯,*K*)^*T*^ and *γ* = (1,⋯,1)^*T*^.

Model 2: There are two factors and genotypes influence one factor. That is, *R* = 2, $\lambda ={\left(\underset{K/2}{\underset{︸}{0,0,\cdots ,0,}}\underset{K/2}{\underset{︸}{\beta ,\beta ,\cdots ,\beta }}\right)}^{T}$, and *γ* = *Bdiag*(*D*_1_, *D*_2_), where *D*_*i*_ = 1_*K*/2_ for *i* = 1, 2.

Model 3: There are five factors and genotypes influence two factors. That is, *R* = 5, $\lambda ={\left(\beta_{11},\cdots ,\beta_{1k},\beta_{21},\cdots ,\beta_{2k},\beta_{31},\cdots ,\beta_{3k},\beta_{41},\cdots ,\beta_{4k},\beta_{51},\cdots ,\beta_{5k}\right)}^{T}$, and *γ* = *Bdiag*(*D*_1_, *D*_2_, *D*_3_, *D*_4_, *D*_5_), where *D*_*i*_ = 1_*K*/5_ for *i* = 1,⋯,5, *k* = *K*/5, $\beta_{11}=\cdots =\beta_{1k}=\beta_{21}=\cdots =\beta_{2k}=\beta_{31}=\cdots =\beta_{3k}=0,$ *β*_41_ = ⋯ = *β*_4*k*_ = −*β* and ${(\beta }_{51},\cdots ,\beta_{5k})=\frac{2\beta }{k+1}(1,\cdots ,k)$.

Model 4: There are five factors and genotypes influence four factors. That is, *R* = 5, $\lambda ={\left(\beta_{11},\cdots ,\beta_{1k},\beta_{21},\cdots ,\beta_{2k},\beta_{31},\cdots ,\beta_{3k},\beta_{41},\cdots ,\beta_{4k},\beta_{51},\cdots ,\beta_{5k}\right)}^{T}$, and *γ* = *Bdiag*(*D*_1_, *D*_2_, *D*_3_, *D*_4_, *D*_5_), where *D*_*i*_ = 1_*K*/5_ for *i* = 1,⋯,5, *k* = *K*/5. $\beta_{11}=\cdots =\beta_{1k}=0,\;\beta_{21}=\cdots =\beta_{2k}=\beta$, *β*_31_ = ⋯ = *β*_3*k*_ = −*β*, ${(\beta }_{41},\cdots ,\beta_{4k})=-\frac{2\beta }{k+1}(1,\cdots ,k)$, and ${(\beta }_{51},\cdots ,\beta_{5k})=\frac{2\beta }{k+1}(1,\cdots ,k)$.

We consider two types of multiple phenotypes. The first one is that all *K* phenotypes are quantitative and the second one is that half phenotypes are quantitative and the other half are qualitative (binary). To generate a qualitative phenotype, we use a liability threshold model based on a quantitative phenotype. A qualitative phenotype is defined to be affected if the corresponding quantitative phenotype is at least one standard deviation larger (smaller) than the phenotypic mean.

In order to ensure the validity of the ceCLC method, we first evaluate the type I error rates of this method. We simulate data under the null hypothesis, that is, *λ* = (0,⋯,0)^*T*^, and consider three different sample sizes, *N* = 1000, 2000, and 3000, under four different models. The type I error rates are evaluated by 10^6^ replications and at the nominal significance levels of 0.001 and 0.0001, respectively. To evaluate power, we simulate data under the alternative hypothesis and consider two different sample sizes, *N* = 3000 and 5000. The powers are evaluated by 1000 replications at the nominal significance levels of 0.05. To better demonstrate the advantages of the ceCLC method, we compare ceCLC with other multiple-traits analysis methods: CLC [33], MANOVA [40], MultiPhen [19], TATES [23], O’Brien [20], and Omnibus. Moreover, we also compare ceCLC with CPASSOC [31], which is an approach that is based on GWAS summary statistics and contains two different tests (Het and Hom). Based on our simulation setting on individual-level data, we can obtain the corresponding summary statistics using linear model for quantitative traits and logistic regression model for binary traits. Notably, the empirical distribution of the Het test statistic is approximated by a gamma distribution, whereas the gamma distribution may not work well when the number of traits is large, in this case, a simulation procedure needs to be used to construct the empirical distribution under the null hypothesis [31]. Since CLC and Het need a simulation procedure to obtain the final p-values, we use 10^5^ replications to evaluate Type I error rates for both of the methods.

### Simulation results

#### (a) Evaluation of type I error rates

Table 1 presents the type I error rates of the ceCLC method for *K* = 20 quantitative phenotypes, and the type I error rates of the other eight methods (CLC, MANOVA, MultiPhen, TATES, O’Brien, Omnibus, Het, Hom) are summarized in S1 Table. The corresponding type I error rates for the case of half quantitative traits and half qualitative phenotypes are recorded in Table 2 and S2 Table. In addition, the type I error rate of the ceCLC method for *K* = 40 are listed in S3 and S4 Tables, and the type I error rates of the other eight methods for *K* = 40 are summarized in S5 and S6 Tables. For 10^6^ replications, the 95% confidence intervals of Type I error rates divided by nominal significance levels of 0.001 and 0.0001 are (0.9381, 1.0619) and (0.8040, 1.1960), respectively; for 10^5^ replications, the corresponding confidence intervals are (0.8041, 1.1959) and (0.3802, 1.6198), respectively.

**Table 1:** The estimated type I error rates divided by the nominal significance levels of the ceCLC method for 20 quantitative phenotypes with 10^6^ replications.

α	Sample	Model1	Model2	Model3	Model4
	1000	0.97	0.97	0.92	0.96
**0.001**	2000	1.05	1.04	1.02	1.05
	3000	0.99	1.03	1.06	0.99
	1000	0.94	0.77	0.71	0.75
**0.0001**	2000	0.89	1.10	0.97	0.95
	3000	0.78	0.86	0.97	0.81

**Table 2:** The estimated type I error rates divided by the nominal significance levels of the ceCLC method for 10 quantitative and 10 qualitative phenotypes with 10^6^ replications.

α	Sample	Model1	Model2	Model3	Model4
	1000	0.99	0.95	0.93	0.98
**0.001**	2000	1.05	0.97	1.05	0.99
	3000	1.05	1.06	1.03	1.06
	1000	1.02	0.90	0.83	0.58
**0.0001**	2000	1.06	0.91	1.09	1.08
	3000	1.10	0.95	1.08	1.04

From Tables 1 and 2 (S3 and S4 Tables), we can see that ceCLC can control the Type I error rate very well, therefore we can conclude that the ceCLC method is a valid test. From S1, S2 and S5, S6 Tables, in summary, we observe that CLC, MANOVA, TATES, O’Brien, Het, and Hom can control type I error rates well, but some of the type I error rates of MultiPhen are slightly inflated.

#### (b) Assessment of powers

**Fig 1:**
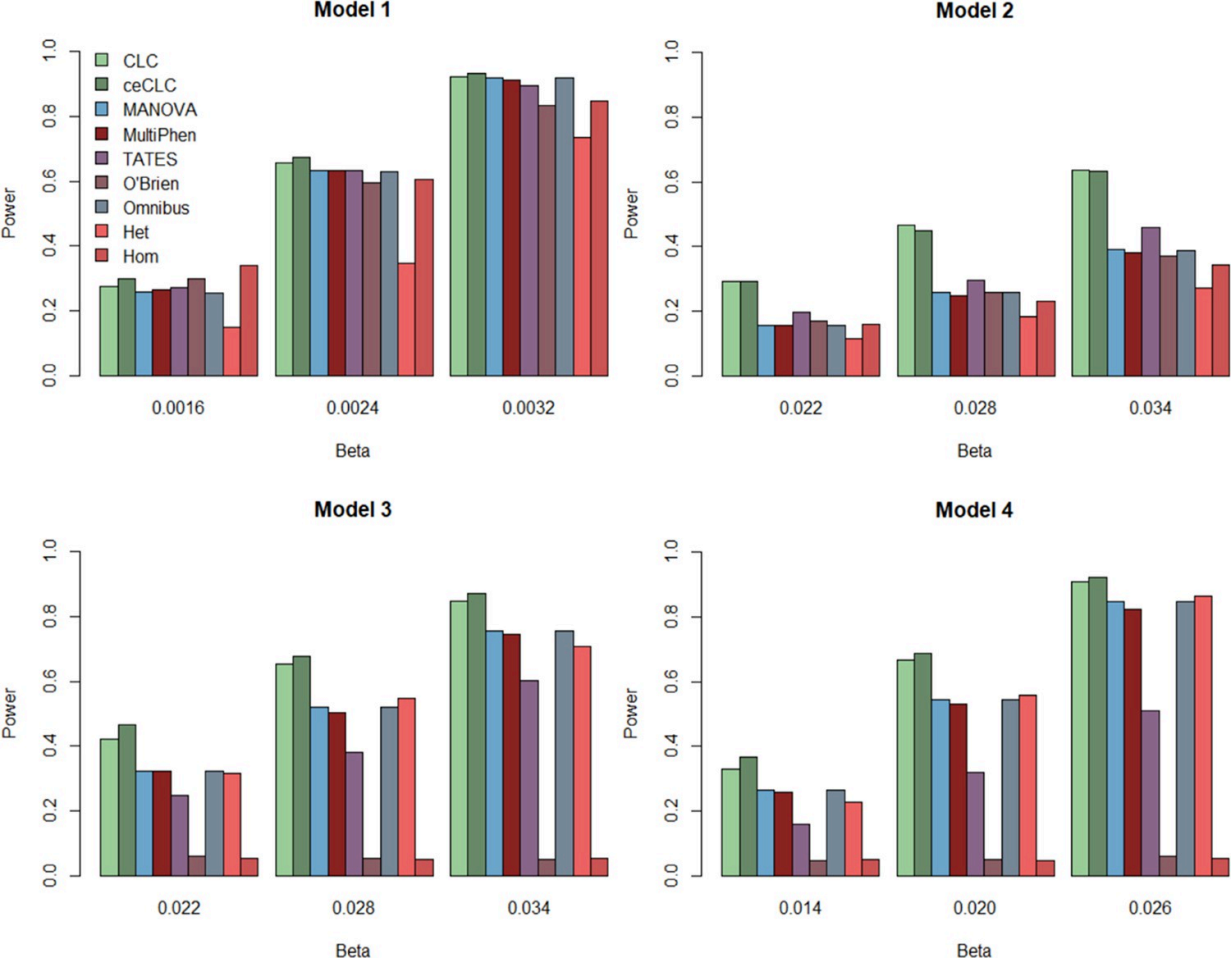
Power comparisons of the nine tests, CLC, ceCLC, MANOVA, MultiPhen, TATES, O’Brien, Omnibus, Het, and Hom with 20 quantitative phenotypes for the sample size of 5000.

**Fig 2:**
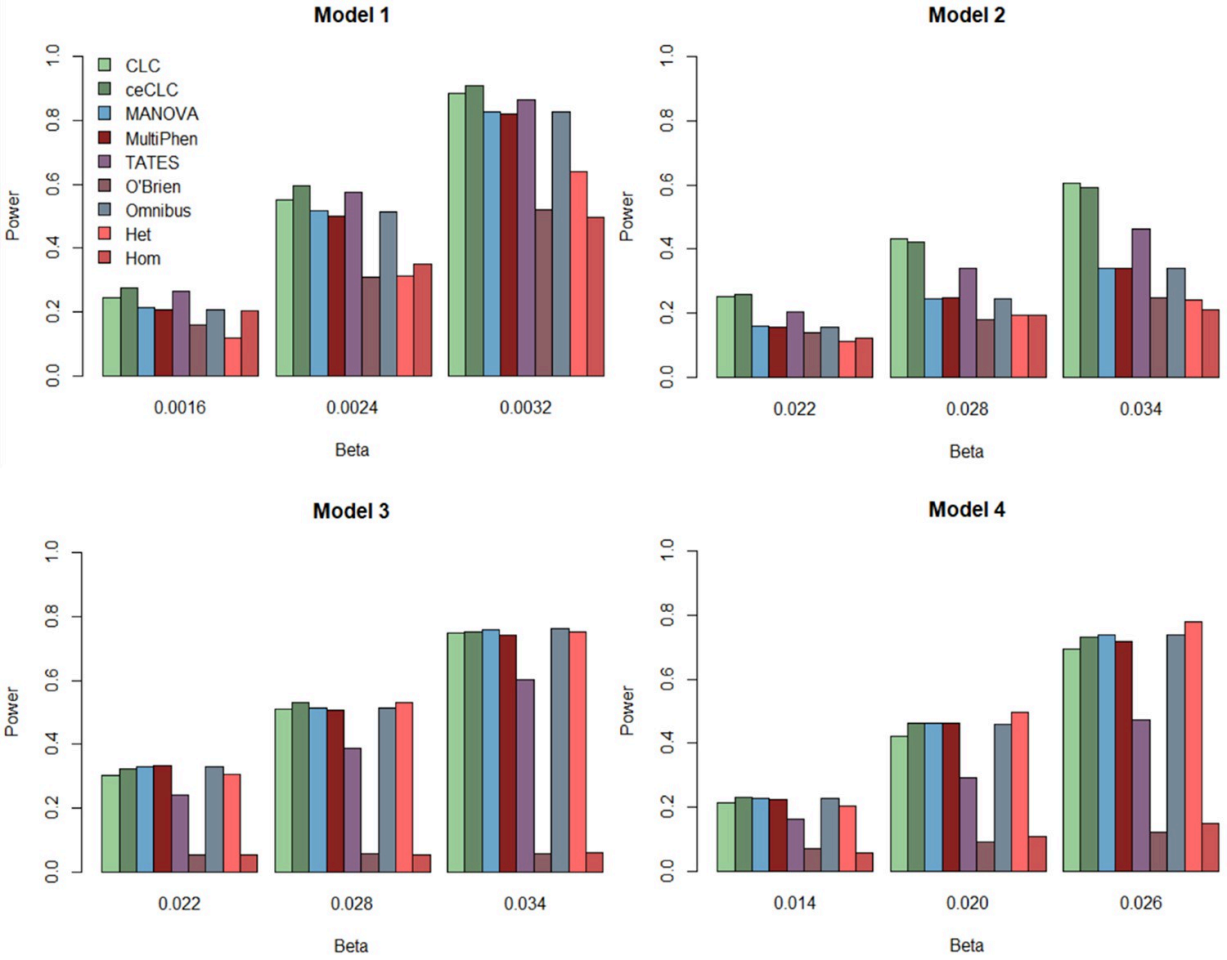
Power comparisons of the nine tests, CLC, ceCLC, MANOVA, MultiPhen, TATES, O’Brien, Omnibus, Het, and Hom with 10 quantitative and 10 qualitative phenotypes for the sample size of 5000.

Fig 1 shows the results of power comparisons for all the nine tests with 20 quantitative phenotypes when the sample size is 5000. From Fig 1, we find that 1) when the variant of interest affects phenotypes with groups (Models 2–4), the ceCLC and CLC methods are more powerful than other methods; 2) the O’Brien and Hom methods are very sensitive to the direction of the genetic effect on the phenotypes. Their powers will decrease dramatically with different directions of the genetic effect on the phenotypes (Models 3 and 4); 3) MANOVA, Omnibus, and MultiPhen show the similar powers in most scenarios. 4) When the effect is homogeneous (Models 1 and 2), Hom is more powerful than Het; when heterogeneity is present (Models 3 and 4), Het performs better than Hom. Fig 2 shows the results of power comparisons for all the nine tests with 10 quantitative and 10 qualitative phenotypes when the sample size is 5000. The general trend of Fig 2 is similar to Fig 1, but the powers of MANOVA, Omnibus, MultiPhen, and Het are higher than those in Fig 1 for Models 3 and 4. S1 and S2 Figs present the results of power comparisons with 40 phenotypes for the sample size of 5000, and all the results of power comparisons for the sample size of 3000 are showed in S3–S6 Figs. In summary, CLC and ceCLC are more powerful than the other methods under most scenarios, and ceCLC is much more computationally efficient than CLC.

## Application to the COPDGene study

Chronic obstructive pulmonary disease (COPD) is a common disease characterized by the presence of expiratory dyspnea due to the excessive inflammatory reaction of harmful gases and particles [41–43]. COPD causes a high mortality and has been reported to be potentially affected by genetic factors [44, 45]. The COPDGene study is a representative multicenter research to detect hereditary factors of this disease [46]. The corresponding dataset of this study was introduced in our previous papers [22, 33], and we use the same processed data as described in Sha et al. [33] for the COPDGene data analysis.

We consider seven quantitative COPD-related phenotypes, containing FEV1, Emphysema, Emphysema Distribution, Gas Trapping, Airway Wall Area, Exacerbation frequency, and Six-minute walk distance. We also consider four covariates which include BMI, Age, Pack-Years and Sex. After removing the missing data, there are 5,430 subjects across 630,860 SNPs left for the analysis. Same with the analysis in [22, 33], the signs of six-minute walk distance and FEV1 were changed, so that the correlations between the 7 phenotypes are all positive. MANOVA, MultiPhen, TATES, and Omnibus are not affected by the sign alignment in phenotypes. CLC and ceCLC are not affected much by the sign alignment. However, O’Brien and Hom are affected very much by the sign alignment [33].

In our analysis, we choose the commonly used genome-wide significant level α = 5×10^−8^ to identify SNPs significantly associated with the 7 COPDrelated phenotypes, Table 3 presents 14 SNPs that are detected by at least one method. All of these 14 SNPs have been reported to be associated with COPD before [47–50]. From Table 3, we can see that MultiPhen detected 14 SNPs; ceCLC, CLC, MANOVA, Omnibus and Het detected 13 SNPs; TATES detected 9 SNPs; O’Brien and Hom only detected 5 SNPs. In Sha et al. [33], single-trait analysis was also performed between each of the seven phenotypes and each of the 14 SNPs. There are four SNPs rs951266, rs8034191, rs2036527, and rs931794, identified by ceCLC, but not identified by any of the single-trait tests. Therefore, these four SNPs are more likely to have pleiotropic effects. Even though we performed the sign alignment, O’Brien and Hom only identified five SNPs. TATES detected 9 SNPs because it mainly depends on the smallest P-value of the seven univariate tests. In summary, the number of SNPs identified by ceCLC is comparable to the largest number of SNPs identified by other tests, which is consistent with our simulation results.

**Table 3:** Significant SNPs and the corresponding p-values in the analysis of COPDGene study.

Chr	Position	Variant identifier	CLC	ceCLC	MANOVA	MultiPhen	TATES	O’Brien	Omnibus	Het	Hom
4	14543149	rs1512282	10^−9^	5.70×10^−11^	1.69×10^−9^	1.03×10^−9^	5.77×10^−9^	7.69×10^−9^	1.82×10^−9^	7.98×10^−10^	7.38×10^−9^
4	14543474	rs1032297	10^−9^	2.39×10^−15^	6.52×10^−14^	7.69×10^−14^	6.22×10^−13^	3.35×10^−10^	7.73×10^−14^	2.34×10^−13^	2.95×10^−10^
4	14547447	rs1489759	10^−9^	3.30×10^−17^	1.11×10^−16^	1.22×10^−16^	2.52×10^−16^	2.61×10^−11^	1.11×10^−16^	1.51×10^−15^	2.24×10^−11^
4	14548573	rs1980057	10^−9^	3.29×10^−17^	6.68×10^−17^	8.14×10^−17^	9.35×10^−17^	3.04×10^−11^	1.11×10^−16^	7.52×10^−16^	2.61×10^−11^
4	14548591	rs7655625	10^−9^	3.30×10^−17^	7.12×10^−17^	9.13×10^−17^	1.64×10^−16^	3.08×10^−11^	1.11×10^−16^	1.38×10^−15^	2.64×10^−11^
15	78882925	rs16969968	10^−9^	4.91×10^−11^	1.32×10^−11^	7.84×10^−12^	2.98×10^−8^	9.75×10^−6^	1.26×10^−11^	1.37×10^−11^	9.40×10^−6^
15	78894339	rs1051730	10^−9^	4.74×10^−11^	1.41×10^−11^	8.16×10^−12^	2.63×10^−8^	8.99×10^−6^	1.35×10^−11^	1.14×10^−11^	8.67×10^−6^
15	78898723	rs12914385	10^−9^	2.57×10^−12^	1.76×10^−12^	1.48×10^−12^	5.14×10^−10^	6.12×10^−8^	1.66×10^−12^	6.26×10^−14^	5.80×10^−8^
15	78911181	rs8040868	10^−9^	5.08×10^−12^	2.74×10^−12^	2.59×10^−12^	2.40×10^−9^	1.53×10^−7^	2.50×10^−16^	1.90×10^−13^	1.46×10^−7^
15	78878541	rs951266	10^−9^	7.03×10^−11^	1.77×10^−11^	1.02×10^−11^	5.17×10^−8^	1.50×10^−5^	1.69×10^−11^	2.80×10^−11^	1.49×10^−5^
15	78806023	rs8034191	10^−9^	8.03×10^−10^	2.14×10^−10^	7.74×10^−11^	1.02×10^−7^	2.13×10^−5^	1.99×10^−10^	3.41×10^−10^	2.06×10^−5^
15	78851615	rs2036527	8.33×10^−10^	1.52×10^−9^	3.99×10^−10^	1.77×10^−10^	1.56×10^−7^	2.65×10^−5^	3.76×10^−10^	5.06×10^−10^	2.58×10^−5^
15	78826180	rs931794	10^−9^	1.18×10^−9^	2.35×10^−10^	9.09×10^−11^	1.18×10^−7^	2.33×10^−5^	2.19×10^−10^	1.07×10^−9^	2.27×10^−5^
15	78740964	rs2568494	3.98×10^−7^	5.02×10^−7^	1.05×10^−7^	4.23×10^−8^	2.88×10^−5^	2.38×10^−3^	9.73×10^−8^	1.26×10^−6^	2.36×10^−3^

The p-values of CLC are evaluated using 10^9^ simulations. The p-values of ceCLC, O’Brien, Omnibus, TATES, MANOVA, MultiPhen, Hom, and Het are evaluated using their asymptotic distributions. The graying out p-values indicate the p-values > 5×10^−8^.

## Discussion

In the medical field, many human complex diseases are often accompanied by multiple correlated phenotypes which are usually measured simultaneously, so jointly analyzing multiple phenotypes in genetic association studies will very likely increase the statistical power to identify genetic variants that are associated with complex diseases. In this paper, based on the existing CLC method [33] and ACAT [34] strategy, we develop the ceCLC method to test association between multiple phenotypes and a genetic variant. We perform a variety of simulation studies, as well as an application to the COPDGene study to evaluate our new method. The results suggest that the ceCLC method not only has the advantages of the CLC method but is also computationally efficient. We compared the running time between ceCLC and CLC in the power comparison. Both methods consider one genetic variant and 20 quantitative phenotypes for 5000 individuals. The running time of ceCLC with 1000 replications on a computer with 4 Intel Cores @3.60 GHz and 16GB memory is about 25s, whereas CLC with 1000 replications and 1000 permutations is about 3min30s. The test statistic of the ceCLC method can be well approximated by a standard Cauchy distribution, so the p-value can be obtained from the cumulative density function without the need for the simulation procedure. Therefore, the ceCLC method is computationally efficient.

In this paper, we apply ceCLC to the COPDGene with seven quantitative COPD-related phenotypes. Recent studies indicate that the pleiotropic effects and genetic heterogeneity are common in the COPD comorbid traits and other immune diseases. For example, Zhu et al. [45] showed evidence of significant positive genetic correlations between COPD and cardiovascular disease-related traits (CVD); Zhu Z et al. [51–53] identified the shared genetic architecture between asthma and allergic diseases [51, 52] and between asthma and mental health disorders [53]. Moreover, pleiotropic effects were found between eight psychiatric disorders [54]. Therefore, ceCLC can also be applied to jointly analyze those phenotypes with shared genetic architecture, thus making it possible to boost statistical power to identify SNPs that were missed by the single-trait genome-wide association analysis. The SNP is more likely to have pleiotropic effect if it was identified by the multiple-trait test but missed by the single-trait test. The detection of SNPs with pleiotropic effects is helpful to promote understanding of the molecular mechanism between co-morbid diseases.

Recent phenome-wide association studies (PheWAS) require more powerful and efficient methods to identify significantly associated SNPs as a large number of phenotypes are collected, the ceCLC method developed in this paper can be applied to PheWAS. However, one limitation of the ceCLC method is that it requires individual-level phenotype data and GWAS summary statistics, where the individual-level phenotypes are used to estimate the trait correlation matrix. Because the individual-level data is often not easily accessible as a result of privacy concerns, we are currently considering a new strategy to extend the ceCLC method applicable to GWAS summary statistics without the requirement for individual-level phenotype data.

## Supporting information

S1 Table The estimated type I error rates divided by nominal significance levels of the other eight methods, CLC, MANOVA, MultiPhen, TATES, O’Brien, Omnibus, Het, and Hom for 20 quantitative phenotypes. (DOCX)

S2 Table The estimated type I error rates divided by nominal significance levels of the other eight methods, CLC, MANOVA, MultiPhen, TATES, O’Brien, Omnibus, Het, and Hom for 10 quantitative and 10 qualitative phenotypes. (DOCX)

S3 Table The estimated type I error rates divided by the nominal significance levels of the ceCLC method for 40 quantitative phenotypes. (DOCX)

S4 Table The estimated type I error rates divided by the nominal significance levels of the ceCLC method for 20 quantitative and 20 qualitative phenotypes. (DOCX)

S5 Table The estimated type I error rates divided by nominal significance levels of the other eight methods, CLC, MANOVA, MultiPhen, TATES, O’Brien, Omnibus, Het, and Hom for 40 quantitative phenotypes. (DOCX)

S6 Table The estimated type I error rates divided by nominal significance levels of the other eight methods, CLC, MANOVA, MultiPhen, TATES, O’Brien, Omnibus, Het, and Hom for 20 quantitative and 20 qualitative phenotypes. (DOCX)

S1 Fig Power comparisons of the nine tests, CLC, ceCLC, MANOVA, MultiPhen, TATES, O’Brien, Omnibus, Hom, and Het with 40 quantitative phenotypes for the sample size of 5000. (PDF)

S2 Fig Power comparisons of the nine tests, CLC, ceCLC, MANOVA, MultiPhen, TATES, O’Brien, Omnibus, Hom, and Het with 20 quantitative and 20 qualitative phenotypes for the sample size of 5000. (PDF)

S3 Fig Power comparisons of the nine tests, CLC, ceCLC, MANOVA, MultiPhen, TATES, O’Brien, Omnibus, Het, and Hom with 20 quantitative phenotypes for the sample size of 3000. (PDF)

S4 Fig Power comparisons of the nine tests, CLC, ceCLC, MANOVA, MultiPhen, TATES, O’Brien, Omnibus, Het, and Hom with 10 quantitative and 10 qualitative phenotypes for the sample size of 3000. (PDF)

S5 Fig Power comparisons of the nine tests, CLC, ceCLC, MANOVA, MultiPhen, TATES, O’Brien, Omnibus, Het, and Hom with 40 quantitative phenotypes for the sample size of 3000. (PDF)

S6 Fig Power comparisons of the nine tests, CLC, ceCLC, MANOVA, MultiPhen, TATES, O’Brien, Omnibus, Het, and Hom with 20 quantitative and 20 qualitative phenotypes for the sample size of 3000. (PDF)
