# Supplementary material for: A computationally efficient clustering linear combination approach to jointly analyze multiple phenotypes for GWAS
Source: PLoS One. 2022 Apr 28;17(4):e0260911. doi: 10.1371/journal.pone.0260911 (PMC9049312; doi:10.1371/journal.pone.0260911)
Supplement: S3 Table — (DOCX) [file pone.0260911.s003.docx]

**S3 Table. The estimated type I error rates divided by the nominal significance levels of the ceCLC method for 40 quantitative phenotypes.**

| $\boldsymbol{\alpha}$ | Sample | Model1 | Model2 | Model3 | Model4 |
| --- | --- | --- | --- | --- | --- |
|  | 1000 | 0.93 | 0.90 | 0.86 | 0.92 |
| 0.001 | 2000 | 0.95 | 0.98 | 0.98 | 0.92 |
|  | 3000 | 1.02 | 0.97 | 1.02 | 1.01 |
|  | 1000 | 0.64 | 0.70 | 0.67 | 0.87 |
| 0.0001 | 2000 | 0.88 | 0.87 | 0.69 | 0.86 |
|  | 3000 | 0.73 | 0.88 | 0.98 | 0.96 |
