# Supplementary material for: A computationally efficient clustering linear combination approach to jointly analyze multiple phenotypes for GWAS
Source: PLoS One. 2022 Apr 28;17(4):e0260911. doi: 10.1371/journal.pone.0260911 (PMC9049312; doi:10.1371/journal.pone.0260911)
Supplement: S4 Table — (DOCX) [file pone.0260911.s004.docx]

**S4 Table. The estimated type I error rates divided by the nominal significance levels of the ceCLC method for 20 quantitative and 20 qualitative phenotypes.**

| $\boldsymbol{\alpha}$ | Sample | Model1 | Model2 | Model3 | Model4 |
| --- | --- | --- | --- | --- | --- |
|  | 1000 | 0.91 | 0.91 | 0.90 | 0.90 |
| 0.001 | 2000 | 1.05 | 1.04 | 1.03 | 0.96 |
|  | 3000 | 1.01 | 1.00 | 1.03 | 1.00 |
|  | 1000 | 0.76 | 0.86 | 0.81 | 0.90 |
| 0.0001 | 2000 | 0.86 | 1.13 | 0.95 | 0.71 |
|  | 3000 | 0.86 | 0.84 | 1.01 | 1.05 |
