# Supplementary material for: A computationally efficient clustering linear combination approach to jointly analyze multiple phenotypes for GWAS
Source: PLoS One. 2022 Apr 28;17(4):e0260911. doi: 10.1371/journal.pone.0260911 (PMC9049312; doi:10.1371/journal.pone.0260911)
Supplement: S5 Table — (DOCX) [file pone.0260911.s005.docx]

**S5 Table. The estimated type I error rates divided by nominal significance levels of the other eight methods (CLC, MANOVA, MultiPhen, TATES, O’Brien, Omnibus, Het, Hom) for 40 quantitative phenotypes.**

| **Model** | **Sample** | $\boldsymbol{\alpha}$ | **CLC** | **MANOVA** | **MultiPhen** | **TATES** | **O’Brien** | **Omnibus** | **Het** | **Hom** |
| --- | --- | --- | --- | --- | --- | --- | --- | --- | --- | --- |
|  | 1000 | 0.001 | 0.98 | 1.05 | 1.16 | 0.93 | 1.00 | 0.78 | 0.62 | 0.92 |
|  |  | 0.0001 | 0.50 | 1.03 | 1.30 | 0.93 | 0.95 | 0.66 | 0.70 | 0.50 |
| **1** | 2000 | 0.001 | 0.94 | 0.94 | 1.03 | 1.00 | 0.82 | 0.84 | 0.84 | 1.01 |
|  |  | 0.0001 | 0.95 | 0.80 | 0.96 | 1.10 | 0.89 | 0.60 | 0.45 | 0.95 |
|  | 3000 | 0.001 | 0.83 | 0.99 | 1.07 | 0.96 | 0.94 | 0.99 | 0.97 | 1.05 |
|  |  | 0.0001 | 0.40 | 1.10 | 1.17 | 0.99 | 0.80 | 1.01 | 0.60 | 0.90 |
|  | 1000 | 0.001 | 0.99 | 1.02 | 1.09 | 0.98 | 1.06 | 0.74 | 0.89 | 1.05 |
|  |  | 0.0001 | 0.50 | 0.75 | 1.26 | 0.85 | 1.05 | 0.45 | 1.10 | 1.12 |
| **2** | 2000 | 0.001 | 0.87 | 0.89 | 0.99 | 0.89 | 0.96 | 0.76 | 1.08 | 0.96 |
|  |  | 0.0001 | 0.75 | 0.60 | 1.01 | 1.00 | 1.08 | 0.60 | 1.24 | 1.15 |
|  | 3000 | 0.001 | 1.13 | 0.97 | 1.03 | 1.03 | 1.00 | 0.93 | 1.02 | 0.98 |
|  |  | 0.0001 | 0.90 | 1.08 | 1.19 | 1.10 | 1.04 | 0.80 | 0.70 | 0.80 |
|  | 1000 | 0.001 | 0.65 | 1.06 | 1.07 | 0.96 | 1.00 | 0.80 | 0.75 | 0.85 |
|  |  | 0.0001 | 0.58 | 1.06 | 1.12 | 1.00 | 1.01 | 0.62 | 0.50 | 1.00 |
| **3** | 2000 | 0.001 | 0.88 | 0.99 | 1.11 | 1.04 | 1.02 | 0.79 | 1.00 | 0.98 |
|  |  | 0.0001 | 1.20 | 1.16 | 1.23 | 0.90 | 1.13 | 1.02 | 0.80 | 1.04 |
|  | 3000 | 0.001 | 1.01 | 1.06 | 1.00 | 0.99 | 0.99 | 0.95 | 1.04 | 0.82 |
|  |  | 0.0001 | 1.12 | 0.85 | 0.97 | 1.00 | 0.80 | 0.85 | 1.30 | 1.10 |
|  | 1000 | 0.001 | 0.89 | 1.04 | 0.98 | 0.89 | 1.02 | 0.60 | 0.97 | 0.83 |
|  |  | 0.0001 | 0.96 | 0.89 | 1.02 | 0.80 | 1.10 | 0.96 | 1.10 | 1.00 |
| **4** | 2000 | 0.001 | 0.74 | 1.05 | 1.09 | 1.06 | 1.02 | 1.00 | 0.96 | 0.99 |
|  |  | 0.0001 | 0.56 | 1.12 | 0.98 | 1.09 | 0.90 | 0.70 | 0.50 | 0.96 |
|  | 3000 | 0.001 | 0.98 | 1.06 | 1.15 | 1.02 | 0.99 | 0.93 | 1.02 | 0.85 |
|  |  | 0.0001 | 1.02 | 0.89 | 1.06 | 1.12 | 1.16 | 0.89 | 1.23 | 1.04 |
