# Supplementary material for: A computationally efficient clustering linear combination approach to jointly analyze multiple phenotypes for GWAS
Source: PLoS One. 2022 Apr 28;17(4):e0260911. doi: 10.1371/journal.pone.0260911 (PMC9049312; doi:10.1371/journal.pone.0260911)
Supplement: S5 Fig — (PDF) [file pone.0260911.s011.pdf]

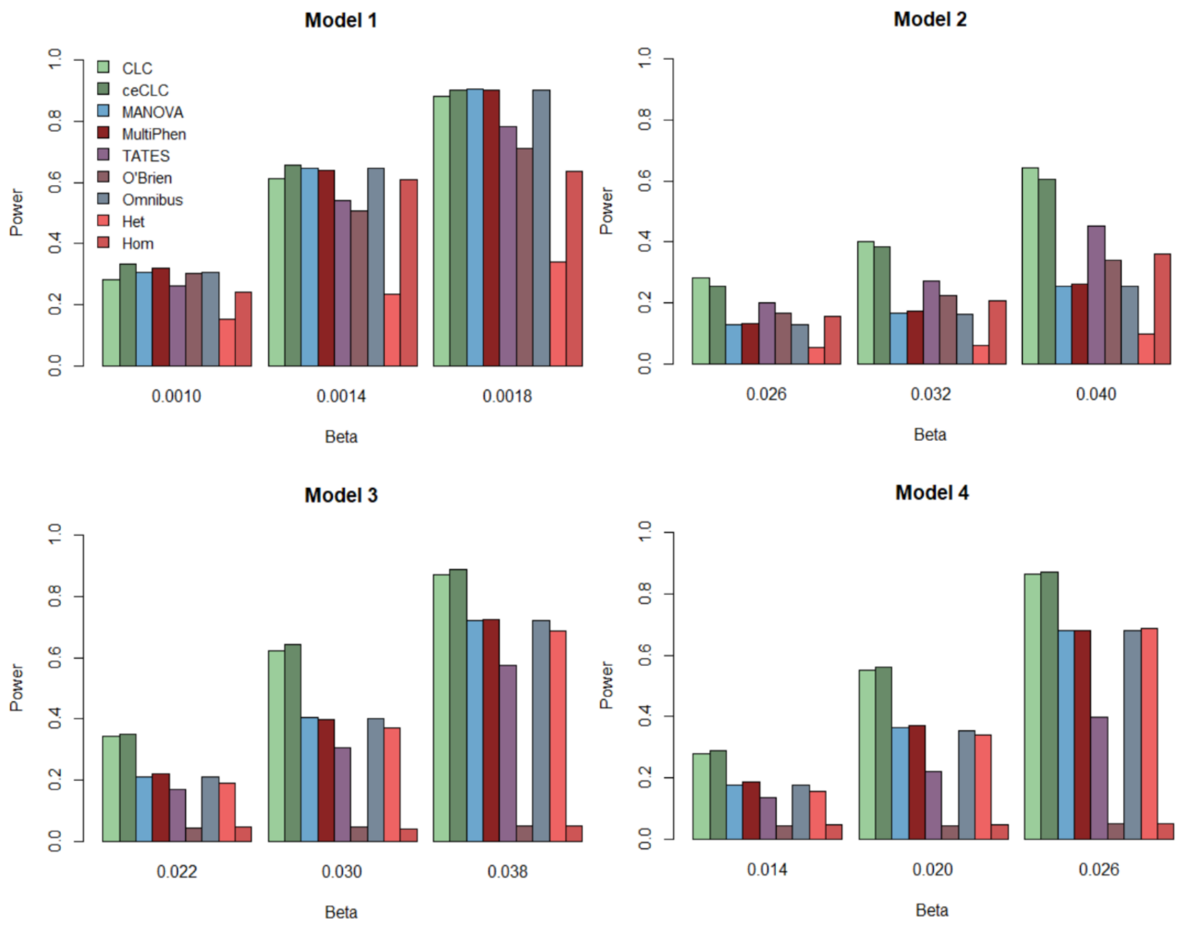

S5 Fig. Power comparisons of the nine tests (CLC, ceCLC, MANOVA, MultiPhen, TATES, O'Brien, Omnibus) with 40 quantitative phenotypes for the sample size of 3000.
